# Diagnostic and Therapeutic Approaches for Heart Failure in Long-Term Survivors of Childhood Cancer

**DOI:** 10.3390/biomedicines12081875

**Published:** 2024-08-16

**Authors:** Consuelo Fernandez-Aviles, Rafael Gonzalez-Manzanares, Soledad Ojeda, Juan C. Castillo, Ainhoa Robles-Mezcua, Manuel Anguita, Dolores Mesa, Manuel Pan

**Affiliations:** 1 Cardiology Unit, Reina Sofía University Hospital, 14004 Córdoba, Spainmd1paalm@uco.es (M.P.); 2 Instituto Maimónides de Investigación Biomédica de Córdoba (IMIBIC), 14004 Córdoba, Spain; 3 Centro de Investigación Biomédica en Red Enfermedades Cardiovasculares (CIBERCV), 28029 Madrid, Spain; 4 Departamento de Ciencias Médicas y Quirúrgicas, Universidad de Córdoba, 14004 Córdoba, Spain; 5 Cardiology Unit, Hospital Universitario Virgen de la Victoria, 29010 Málaga, Spain; 6 IBIMA-Plataforma BIONAND, Universidad de Málaga, 29071 Málaga, Spain

**Keywords:** heart failure therapy, cardiotoxicity, long-term survivors, childhood cancer

## Abstract

The improvement in survival rates in pediatric malignancies has led to an increase in the number of cancer survivors who are at risk of developing cardiotoxicity and heart failure. Cardiac dysfunction in these patients can occur asymptomatically, and the diagnosis in a symptomatic phase is associated with reduced treatment response and worse prognosis. For this reason, it is essential to establish protocols to follow up on these patients and identify those at risk of cardiotoxicity in order to start early and effective therapies. This review aims to summarize the latest findings in the diagnosis and treatment of cancer therapy-related cardiac disease in long-term survivors of childhood cancer, with a focus on heart failure.

## 1. Introduction

Antineoplastic therapies have evolved during the last decades, with the resulting improvement in pediatric cancer survival rates, which are over 80% at 5 years from diagnosis [1]. This inevitably leads to an increase in the number of adult childhood cancer survivors (CCS). Nevertheless, mortality rates in CCS are higher when compared to the general population. Recurrence of the main tumor is the first cause of death in this population, followed by second malignancies and cardiac disease [2,3]. Acute lymphoblastic leukemia (ALL) is the most common type of childhood malignancy. Thanks to the advances in antineoplastic therapies, the five-year survival rate has improved from 50% in the 1970s to around 90% in the current time [4]. This has led to an increase in the number of childhood leukemia survivors who are at risk of developing side effects of these treatments, even in the long term. Anthracyclines are one of the most effective therapies in ALL; nevertheless, their use involves a risk of cardiovascular disease (CVD) during follow-up [5]. Clinical manifestations of cardiotoxicity can be diverse, with congestive heart failure (HF) due to myocardial dysfunction being the most common. HF symptoms can appear years after treatment, but CVD can previously develop in a subclinical way [6]. Diagnosis at a symptomatic stage can limit the effectiveness of treatments and worsen their prognosis. This points out the need to establish follow-up protocols for all CCS exposed to anthracyclines in order to identify those at risk of CVD and implement early treatment. This can be a real challenge for public health systems because close and lifelong follow-ups of all these patients would be an unbearable economic and care burden. The purpose of this review is to gather the latest developments in the diagnosis and treatment of cardiotoxicity in CCS.

## 2. Prevalence of Heart Failure in Long Survivors of Childhood Cancer

### 2.1. Prevalence of Symptomatic Heart Failure in Long Survivors of Childhood Cancer

Cardiotoxic effects on long-term CCS treated with anthracyclines started being noticed during the 1990s. From then on, long-term cardiac effects were thoroughly evaluated in large cohorts of survivors that often included healthy siblings as controls. In addition, the increased risk of HF was confirmed in population-based registries. After gathering the information from these studies, it can be concluded that the rates of symptomatic HF in CCS increase with time since diagnosis and treatment exposure, with those patients receiving higher doses of anthracyclines and chest radiotherapy being at higher risk. Overall, the risk of symptomatic HF ranges between 1 and 5% (Table 1). In The Dutch Childhood Oncology Group Long-Term Effects After Childhood Cancer study, Feijen et al. reported an incidence of HF of 2% after a mean follow-up of 19 years and 4.4% 40 years after diagnosis [7]. The Childhood Cancer Survivor Study included over 10,000 childhood cancer survivors diagnosed in the United States and Canada and a control group of healthy siblings. The investigators found a cumulative incidence of HF of 4.8% by 45 years of age [8]. The British Childhood Cancer Survivor Study gathered 34.489 cancer survivors diagnosed before the age of 15 between 1940 and 2006 in the United Kingdom. They were followed for a mean of 31.4 years and showed a six times higher risk of HF mortality than expected [9]. The Adult Life after Childhood Cancer in Scandinavia analyzed the prevalence of HF admission in a cohort of 32.308 cancer survivors after a follow-up of 10 years and found a 5.2 times higher risk compared to a control group of similar age and sex [10]. The group of the German Childhood Cancer Registry examined a cohort of 951 patients diagnosed between 1980 and 1990 and found an incidence of HF of 1.2% [11].

### 2.2. Prevalence of Left Ventricular Systolic Dysfunction in Long Survivors of Childhood Cancer

The previously mentioned studies were focused on the development of symptomatic HF. Nevertheless, there is also evidence that CCS often presents asymptomatic left ventricular systolic dysfunction (LVSD). The prevalence of LVSD ranges from 4% to 16.3% [12,13,14,15]. The reported rates are partially conditioned by the chosen echocardiographic measurement to define LVSD, with left ventricular fractional shortening being more frequently used in initial studies and left ventricular ejection fraction (LVEF) in more recent ones. The prevalence of asymptomatic LVEF-based LVSD in different cohorts is summarized in Table 1. The main limitation of these studies was the cross-sectional design, which often impeded differentiating those LVSD cases that occurred in the acute setting from those that developed over time. To date, there are scarce data from longitudinal studies that included serial echocardiographic assessment. Rathe et al. reported a deterioration of LVEF over time in 80 ALL children survivors treated with anthracycline doses of 300 mg/m^2^ or less [18]. Accordingly, Lipshultz et al. studied 499 serial echocardiograms from 115 long-term survivors of ALL treated with Doxorubicin and found a progressive reduction in left ventricular fractional shortening up to 12 years after therapy [19]. The impairment in contractility was accompanied by a progressive reduction in ventricular mass. Despite 2D echocardiogram being the most commonly used method to measure LVEF in routine practice, the use of 3D echocardiography for serial LVEF measurement is a promising tool to reduce temporal variability and to enhance our understanding of left ventricular systolic function dynamics in long-term CCS [20].

## 3. New Tools for the Diagnosis of Cardiovascular Disease

### 3.1. Global Longitudinal Strain

Screening for cardiac dysfunction in cancer survivors has been mainly based on the measurement of left ventricle ejection fraction (LVEF) in two dimensions by echocardiography, and until recently, this was the sole parameter used to define ventricular dysfunction due to cardiotoxicity. There are other echocardiographic parameters, such as LVEF, measured in three dimensions or GLS, that are gaining popularity due to their better correlation to LVEF measured by cardiac magnetic resonance (CMR), which is considered the gold standard technique [21].

Aiming to establish a new definition of cardiotoxicity, the latest European Guidelines on Cardio-Oncology bring together cardiac injury, cardiomyopathy, and HF as possible presentations of the global term cancer therapy-related cardiac dysfunction (CTRCD) [22]. CTRCD is classified according to the presence of symptoms and the measurement of LVEF and GLS. Symptomatic CTRCD is a clinical syndrome consisting of HF cardinal symptoms and signs, and it is divided into distinct phenotypes based on LVEF: ≤40% = HFrEF (HF with reduced EF); 41–49% = HFmrEF (HF with mildly reduced EF); ≥50% = HFpEF (HF with preserved EF). Asymptomatic CTRCD is defined by either an LVEF reduction to <40%, an LVEF reduction by ≥10% to an LVEF of 40–49%, or <10% to an LVEF of 40–49% and a decline in GLS > 15% or a rise in cardiac biomarkers, or a decline > 15% in GLS, and/or a rise in cardiac biomarkers to an LVEF ≥ 50%.

**Figure 1:**
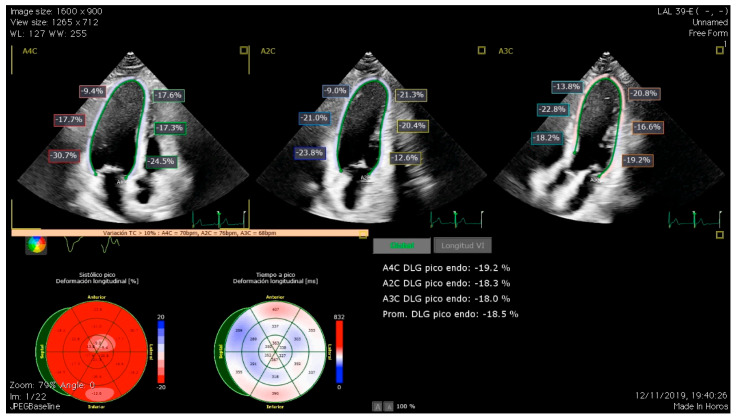
Global longitudinal strain (GLS) assessment in a long-term survivor of childhood cancer using automated software (QLAB 15.0., Philips Medical Systems, Andover, MA, USA). The upper panels show automatic segment quantification of GLS by speckle tracking in the four-chamber, two-chamber, and three-chamber views. The lower panels show GLS values in the bull’s eye plot and average values of GLS.

The importance of GLS relies on its ability to detect subclinical dysfunction, given its high sensitivity in the detection of changes in the deformation of longitudinal myocardial fibers (Figure 1). There are multiple studies that have proven its value in the assessment of cardiotoxicity [23]. In line with this, our group recently investigated the role of GLS in detecting subclinical LVSD in a cohort of long-term childhood leukemia survivors, finding abnormal GLS values in up to 26% of the survivors [14]. Later, we evaluated LV diastolic function using conventional parameters and left atrial strain (LAS), finding an association between incremental doses of anticancer treatments and LAS impairment and displaying LAS as a potential early marker of diastolic dysfunction in these patients [24]. LAS is a relatively novel echocardiographic parameter with good sensitivity and specificity in the assessment of LV diastolic function and has shown a good correlation with invasive techniques for the assessment of filling pressures [25]. All the evidence previously exposed on the diagnosis of CTRCD revolves around the evaluation of LV function. Nevertheless, patients exposed to chemotherapy are not exempt from suffering right ventricle (RV) dysfunction. Evaluation of RV systolic function can be challenging due to its intricate geometry, and, in this aspect, RV strain has become a useful tool as it showed a better correlation with RV ejection fraction than other traditional parameters [26]. The role of RV strain measurement and the significance of RV impairment in terms of prognosis is not clearly established. Our research group reported a decrease in CCS RV strain that was inversely associated with modifiable ca and smoking habit [27]. Abnormal values of RV strain have also been reported in other cohorts of long-term CCS. However, further longitudinal studies are needed to clarify the prognostic value of this finding [28].

### 3.2. Cardiac Magnetic Resonance

**Figure 2:**
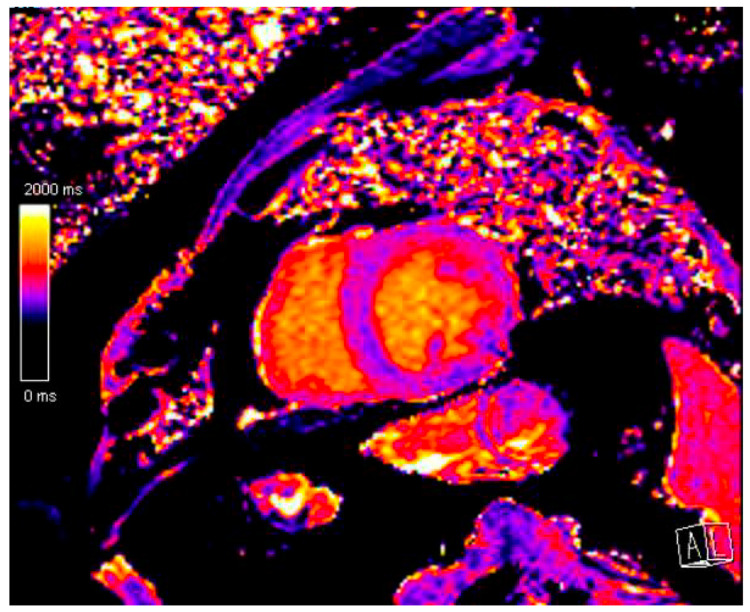
Native T1 Mapping in a long-term survivor of childhood cancer with mildly reduced ejection fraction showing slightly elevated values. Native T1 Mapping values are determined by the combination of myocytes and extracellular volume signals. An increase can be found in the context of edema or an increase in the interstitial space.

As was mentioned before, CMR is the gold standard technique for determining LVEF in patients undergoing chemotherapy and during follow-up. The accuracy, precision, and reproducibility of CMR make it an essential tool for CTRCD diagnosis. Nevertheless, the cost of CMR and its reduced availability are important limitations that preclude its use as a routine test [21]. Apart from quantifying LVEF, CMR enables tissue characterization, which constitutes a promising tool for the early diagnosis of CTRCD, both in the acute and chronic setting. In the acute setting, myocyte apoptosis and atrophy are the main mechanisms of AC-related cardiac damage, more than fibrosis, and there is a component of edema in the early stages of cardiotoxicity [29]. There are animal models that have shown how T2 relaxation values are increased during the first weeks after administrating Doxorubicin, before either T1, extracellular volume, or LVEF are affected. In animals that discontinued the therapy when the alterations in T2 were found, the rest of the parameters remained unaffected, whereas, in the ones who continued, LVEF was reduced, and T1 and extracellular volume increased [30]. This suggests that T2 values could be a marker of reversible myocardial damage in these patients. However, there is a lack of evidence to support the implementation of tissue characterization to assist clinical decision-making in cardio-oncology. In a prospective cohort of cancer breast women receiving anthracycline and trastuzumab therapy, CMR myocardial tissue characterization revealed transient changes due to edema and inflammation that were not associated with CTRCD [31]. In the long-term follow-up, T1 mapping and extracellular volume measurements of diffuse interstitial fibrosis have been reported to be abnormal in a cohort of adult cancer survivors [32] (Figure 2). There is a recent meta-analysis that reviewed the evidence supporting native myocardial T1 mapping as a measure to detect early myocardial affection in patients treated with AC [33]. Nine studies were finally included; in all of them, patients were exposed to AC-based treatments with concomitant radiotherapy in one of them. All patients showed a significant increase in native myocardial T1 mapping compared to baseline and healthy controls. It is important to highlight that in all patients, LVEF remained within normal limits, suggesting that native myocardial T1 mapping may have had an important role in detecting CTRCD in a subclinical phase before functional alterations took place.

The question remains on whether myocardial fibrosis measured with T1 mapping and/or extracellular volume will be a valid surrogate of adverse prognosis and overt heart failure, as has been described in other cardiac conditions [34]. Apart from CTRCD, chemotherapy can cause other types of cardiac toxicities, such as pericardial disease, myocarditis, coronary disease, or valvular heart disease, in which CMR has a vital role in differential diagnosis [35].

### 3.3. Blood Biomarkers

In the acute setting, cardiac biomarkers are a useful tool in the risk stratification, diagnosis, and follow-up of CTRCD, natriuretic peptides (NPs) and cardiac troponin being the most supported by the literature. Elevated baseline levels of NPs can identify patients at higher risk of developing cardiotoxicity, allowing for the selection of those patients who would benefit from closer monitoring or even cardioprotective treatments [36,37,38]. However, the utility of cardiac biomarkers for the detection of LVSD is limited in the chronic setting [39,40]. With respect to troponin, this can be easily explained by the fact that it is abruptly released when myocardial injury occurs, coinciding with cardiotoxic treatment administration years before. For NPs, the most plausible explanation is the high proportion of mild LVSD, with no functional or hemodynamic repercussion among those survivors with LVSD, limiting its application for screening in asymptomatic CCS [14]. Recently, Leerink J.M. et al. reported that the addition of N-terminal pro-B-type NP and high sensitivity troponin T levels to clinical characteristics (sex, age, anthracycline dose, chest radiotherapy exposure) was superior to clinical characteristics alone to rule out LVSD in childhood cancer survivors of the DCOG LATER study, opening up a path for multiparametric risk stratification in CCS [41]. Circulating microRNAs (cmiRNAs) are emerging as new biomarker candidates for acute CTRCD monitoring, but to our knowledge, their utility in the cardiac surveillance of long-term survivors has not been explored to date [42]. These epigenetic regulators, through their influence on messenger RNA expression and translation, are implicated in multiple pathological pathways, such as AC-induced cardiotoxicity. They can be found in the cytoplasm but can also be released into the bloodstream, where they remain stable due to their resistance to nucleases. The latter makes cmiRNAs, an attractive biomarker in CTRCD [43]. There are animal models that have studied the role of cmiRNAs in cardiotoxicity. In mice exposed to doxorubicin, miR-34a showed a dose-dependent and early elevation (6 days after treatment) that was correlated with cardiac troponin alteration and preceded clinical manifestations of cardiac dysfunction. Also, this increase was significantly higher in mice that developed cardiotoxicity in comparison with those that did not, suggesting the role of miR-34a as an early cardiac damage marker [44,45]. miR-1 and miR-133a showed an increase in rats 24 h after a single dose of AC, whereas in mice, miR-1 was elevated after four doses of doxorubicin [46,47]. Regarding the role of miRNAs in AC-induced toxicity in humans, Leger et al. conducted a pilot study in children receiving AC and compared them with a control cohort of patients receiving non-cardiotoxic chemotherapy. Also, they correlated the cmiRNAs alterations with high-sensitivity troponin levels as a known marker of cardiac damage. Blood samples were gathered at baseline and 6, 12, and 24 h after the last dose of chemotherapy. They reported an overall increase in cmiRNAs in the AC group compared to the controls. Specifically, miR-29b and miR-499 were elevated in children with acute AC-related cardiac damage, evidenced by cardiac troponin increase, and their increase was proportional to the AC dose [42]. Even though the role of cmiRNAs as cardiac damage biomarkers has been settled, there is a need for further evidence regarding the development of clinical cardiotoxicity. Table 2 shows some of the cmiRNAs previously assessed in clinical studies that evaluated the utility of these biomarkers in diagnosing heart failure or acute cardiotoxicity, which might be of interest in future studies investigating their potential role in detecting CTRCD in long-term CCS.

## 4. Cellular Senescence and Cardiotoxicity

Cellular senescence is a fundamental aging process that can contribute to the pathophysiology of multiple chronic diseases and age-related dysfunction. It can be triggered by several factors, such as DNA damage, due to anticancer therapy. Thus, CCS is thought to present an accelerated aging-like type phenotype that favors the development of cardio-metabolic diseases [54]. It has been shown that chemotherapy, including anthracyclines, can induce a senescent response in myocardial cells, including fibroblasts, endothelial cells, and cardiomyocytes [55]. Among the underlying mechanisms are mitochondrial dysfunction and an increase in oxygen-reactive species [56]. Also, the development of a senescence-associated secretory phenotype (SASP) and the production of proinflammatory cytokines, chemokines, and matrix metalloproteinases seems to be related to this process [57]. The accumulation of senescence and the inflammation provoked by these substances can lead to myocardial remodeling and LV dysfunction. This is illustrated in the study carried out by Linders et al. [58]. The investigators used engineered heart tissues that were sequentially exposed to Doxorubicin. In the end, levels of senescence markers, SASP markers, mitochondrial function, as well as systolic and diastolic function parameters were measured. Elevated levels of senescence genes and proteins, such as p16 and p53, were observed in the tissues, and signs of contractile and mitochondrial dysfunction were found, supporting the senescence pathway as an important mechanism of LV dysfunction related to AC. The development of senolytic drugs, targeted specifically against senescent cells, might be a promising strategy for preventing and treating chronic diseases in CCS [59]. Demaria et al., using a transgenic mice model, showed that Doxorubicin-related cardiac dysfunction was associated with senescent cells and that the elimination of these cells (marked with p16) prevented this alteration [57].

## 5. Treatment of Heart Failure in Cancer Survivors

To prevent CRTCD from appearing, it is vital to establish primary prevention strategies and to have the support of a multi-disciplinary team when facing complex clinical scenarios. Cardiovascular risk factors should be managed according to the ESC Guidelines on Cardiovascular Disease Prevention [60]. Renin–angiotensin–aldosterone system blockers, beta-blockers, and mineralocorticoid receptor antagonists have been evaluated in the prevention of AC-related cardiotoxicity and appear to prevent LVEF reduction in the acute setting. The prevention of cardiac dysfunction during adjuvant breast cancer therapy (PRADA) trial used CMR to compare LVEF changes in breast cancer patients under cardioprotective treatment versus placebo, showing that candesartan initiated before chemotherapy reduced the decline in LVEF [61]. A retrospective study of 920 patients treated with AC found that those treated with beta-blockers for other reasons had a lower incidence of HF [62]. Current ESC guidelines state that angiotensin-converting enzyme inhibitors (ACE-I) or angiotensin receptor blockers (ARB) and beta-blockers should be considered for primary prevention in high- and very high-risk patients receiving AC and/or anti-HER2 therapies (class IIa, level B) or other therapies that can potentially cause HF (class IIa, level C). Dexrazoxane, through its binding to Topoisomerase 2B, has proven protective activity against AC, and it is recommended in adult patients receiving a high total cumulative AC dose for curative treatment and in patients with high and very high CTRCD risk [22,63]. In the event that CTRCD appears, whether symptomatic or asymptomatic, a multi-disciplinary discussion seems necessary in order to decide if chemotherapy should be continued or not. AC should be discontinued in cases of severe symptomatic CTRCD and temporally interrupted in patients with moderate symptomatic CTRCD or severe asymptomatic CTRCD. In patients with mild symptomatic CTRCD, the decision should be individualized and decided in conjunction. Guideline-based treatment of HF (with ACE-I/ARB or angiotensin receptor-neprilysin inhibitor, a beta-blocker, a mineralocorticoid receptor antagonist, and a sodium-glucose co-transporter 2 inhibitor) should be initiated and titrated in patients with symptoms or asymptomatic moderate or severe CTRCD. The decision to interrupt or discontinue AC and, moreover, the decision to restart it in patients who have recovered from CTRCD must be carefully considered. Unfortunately, evidence to assist in this decision-making remains scarce. A small study studied breast cancer patients under treatment with Trastuzumab who developed mild CTRCD and initiated HF therapy. It showed that among patients who did not interrupt chemotherapy, there were no cases of LVEF < 40% or clinical HF and LVEF, CMR-measured LV volumes, or markers of edema and fibrosis, and quality-of-life measures showed no differences compared to a control group that interrupted Trastuzumab [64]. In the context of recovered cardiac dysfunction with the necessary restart of chemotherapy, some modifications must be taken into consideration. Doses and infusion rates should be adjusted, with slow infusion rates preferred [65]. Also, there are liposomal AC preparations, such as liposomal doxorubicin, that have been shown to be less cardiotoxic compared to traditional presentations and should be considered. Finally, pre-treatment with dexrazoxane before AC cycles should be considered in those patients with a very high risk of CTRCD, including patients with previous HF or altered LVEF, in which treatment with AC is fundamental [66].

**Figure 3:**
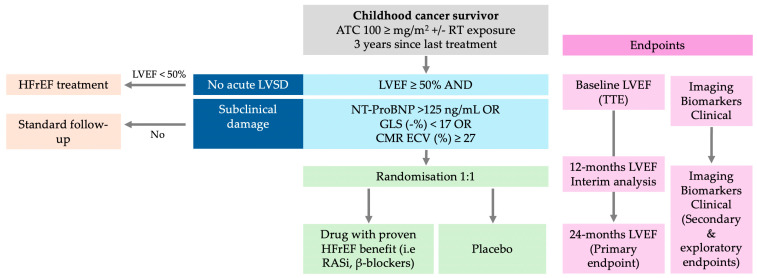
Proposed Randomized Clinical Trial protocol for heart failure prevention in long-term survivors of childhood cancer. ATC: anthracyclines; CMR: cardiac magnetic resonance; GLS: global longitudinal strain; HFrEF: heart failure with reduced ejection fraction; LVEF: left ventricular ejection fraction; LVSD: left ventricular systolic dysfunction; RT: radiotherapy; TTE: transthoracic echocardiography.

There is little evidence of the benefits of cardioprotective treatment in long-term cancer survivors. The recently published PREVENT-HF study randomized 196 patients exposed to AC at least two years before inclusion with an LVEF > 50% and assigned them to carvedilol or placebo for two years. Even though carvedilol demonstrated safety, the results regarding the primary endpoint (reduction in left ventricular wall thickness–dimension ratio Z score) were neutral [67]. In this trial, only high-risk survivors exposed to doses of anthracyclines greater than 250 mg/m^2^ equivalents of doxorubicin were included, a target population in whom carvedilol treatment could be hypothesized to exert a greater benefit. However, the inclusion of an LVEF > 50% as an eligibility criterion could have paradoxically implied the recruitment of participants with lower susceptibility to anthracycline cardiotoxicity since they did not present CTRCD in the acute setting despite the high anthracycline dose. For this reason, clinical trials considering biomarkers of subclinical cardiotoxicity as inclusion criteria, such as a decrease in GLS, the presence of diffuse fibrosis detected by CMR, or abnormal blood biomarkers levels, could increase the probability of demonstrating a beneficial treatment effect of heart failure neurohormonal therapy in long-term cancer survivors [68] (Figure 3).

Another frequent scenario is the development of cardiac arrhythmias during cancer treatment. Specifically, atrial fibrillation (AF) shows an incidence between 2 and 16% in patients under antineoplastic therapy, and the pathophysiology in this context is complex. Many factors can predispose AF in these patients, such as advanced age, subjacent inflammation, metabolic disorders, and some anticancer therapies of cancer surgery-related AF [69]. The occurrence of AF in this context is associated with a two-fold risk of thromboembolism and a six-fold risk of HF [70]. Diagnosis and management of AF, as well as anticoagulation indications, should follow current guidelines and recommendations, considering the frequent interactions between rhythm control drugs and antineoplastic therapies and the complexity of stratifying stroke/bleeding risk in some of these patients [71].

As was previously mentioned, there is growing interest in senolytic therapies. It has been observed that senescent cells expressed antiapoptotic pathways that promoted their survival and apoptosis resistance and that the inhibition of these pathways could lead senescent cells to apoptosis but not proliferative cells [72]. These findings have set the ground for the development of senolytic drugs. Lerida-Viso et al. used the senolytic navitoclax, a Bcl-2 family protein inhibitor, in a mice model to eliminate doxorubicin-related cardiac dysfunction. Mice treated with navitoclax showed fewer senescent cells and no decline in LV shortening fraction compared to mice that received doxorubicin and no senolytic drug [73]. Nevertheless, there is concern regarding the senolytic apoptotic mechanism of action, given the limited regenerative potential of cardiomyocytes. Additional studies are needed to evaluate if this could lead to cardiac dysfunction and, eventually, cardiomyopathy and HF. There are senomorphic agents that interfere with senescent signaling pathways that modulate their expression without producing apoptosis. One example is the widely used metformin, which, through mechanisms that are not fully understood, has shown cardioprotective effects in animal models treated with AC and suppression of senescence in myocardial cells [74,75,76]. Although evidence on senolytic and senomorphic therapies is limited to in vitro studies, these point out these drugs as potential resources to prevent or reverse cardiotoxicity.

## 6. Future Research Direction

The initiated development in cardio-oncology should move toward the creation of specialized units focused on cardiotoxicity as a dynamic process that starts with cancer treatment and accompanies the patients throughout life. In the case of long-term survivors, more research is needed to improve risk stratification and to prove the efficacy of screening and follow-up programs. In addition, dedicated randomized trials with appropriate eligibility criteria and endpoints to evaluate the effect of cardioprotective treatments or senolytic therapies must be carried out.

## 7. Conclusions

Advances in childhood cancer have led to a great improvement in patients’ survival. Nevertheless, cancer treatment-related cardiotoxic effects become a threat to their survival and quality of life during adulthood. This review has gathered the latest advances in the diagnosis and treatment of HF in this population. Regarding diagnosis, the scope is shifting from the LVEF-centric view toward novel tools that allow for the detection of subclinical cardiac dysfunction. With respect to treatment, dedicated randomized controlled trials of HF drugs are needed. Moreover, based on the fact that chemotherapy can activate cell senescence and accelerate aging pathways, senolytic drugs show promise as cardioprotective therapies.

## Figures and Tables

**Table 1:** Heart failure data from selected cancer childhood survivor studies.

Study	*n*	Follow-Up (Years)	Symptomatic HF	LVSD (EF < 50%)	Subclinical LVSD (GLS)
Feijen, 2019 [7]	5845	19.9 (5–50)	2% ^a^	-	-
Armstrong, 2013 [8]	10,724	25.6 (7–39)	4.8%	-	-
Faber, 2018 [11]	951	28.4 (23–36)	1.2%	-	-
Armstrong, 2015 [12]	1820	23 (10–48)	-	5.8%	28.0%
Massey, 2020 [13]	104 ^b^	17.2 ± 5.6	-	16.3%	32.7%
Gonzalez-Manzanares, 2022 [14]	90	18 (11–26)	1.1%	12.2%	26.6%
Christiansen, 2014 [15]	125	20.4 ± 8.6	-	4%	-
Christiansen, 2016 [16]	191	21.6 ± 7.9	-	-	28%
Niemelä, 2021 [17]	90	8.1 (6–13)	-	-	11.1%

^a^ A total of 4.4% 40 years after cancer diagnosis. ^b^ This study included 104 hematopoietic stem cell transplantation survivors, of whom 77 were cancer survivors. EF: ejection fraction; GLS: global longitudinal strain; HF: heart failure; LVSD: left ventricular systolic dysfunction.

**Table 2:** Potential miRNA for the detection of cardiotoxicity in long-term survivors of childhood cancer.

Study	Sample Size	miRNA	Context	Interpretation
Corsten, 2010 [48]	In total, 33 patients admitted with acute HF and 34 healthy controls	miR-499	Acute HF	miR-499 was significantly elevated (2-fold) as compared with control subjects
Goren Y., 2012 [49]	In total, 30 stable chronic HFrEF patients and 30 controls	miR-423-5p, miR-320a, miR-22, and miR-92b	Chronic HFrEF	A score based on the serum levels of these four miRNAs predicted HF and was correlated to prognostic parameters such as elevated NPs or QRS width.
Zhang J., 2017 [50]	Hospitalized patients undergoing coronary angiogram, radiofrequency ablation, or cardiac resynchronization therapy: 80 patients with LVEF < 50% were compared to 40 with LVEF ≥ 50%	miR-21	HFrEF	miR-21 levels in patients with LVEF < 50% were higher than in control subjects and were correlated with NPs and prognosis
Lakhani H.V., 2021 [51]	A total of 17 women with breast cancer	miR-34a	Acute cardiotoxicity	miR-34a increased after the completion of anthracycline treatment and was correlated with a rise in troponin I but not with LVEF
Feng Q., 2021 [52]	A total of 72 breast cancer patients	miR-130a	Acute cardiotoxicity	miR-130a increment during treatment was more pronounced in patients with cardiotoxicity
Frères P., 2018 [53]	A total of 45 breast cancer patients	miR-423	Acute cardiotoxicity	miR-423 rise after the initiation of therapy was correlated with the decline in LVEF at follow-up

HF: heart failure; HFrEF: Heart failure with reduced ejection fraction; LVEF: left ventricular ejection fraction; NPs: natriuretic peptides.

## Data Availability

Not applicable.
